# Short-range contributions of local sources to ambient air

**DOI:** 10.1093/pnasnexus/pgac043

**Published:** 2022-04-14

**Authors:** Elena S Gusareva, Nicolas E Gaultier, Akira Uchida, Balakrishnan N V Premkrishnan, Cassie E Heinle, Wen J Phung, Anthony Wong, Kenny J X Lau, Zhei H Yap, Yanqing Koh, Poh N Ang, Alexander Putra, Deepa Panicker, Jessica G H Lee, Luis C Neves, Daniela I Drautz-Moses, Stephan C Schuster

**Affiliations:** Singapore Center for Environmental Life Sciences Engineering (SCELSE), Nanyang Technological University, 60 Nanyang Drive, Singapore 637551, Singapore; The Asian School of the Environment, Nanyang Technological University, 62 Nanyang Drive, Singapore 637459, Singapore; Singapore Center for Environmental Life Sciences Engineering (SCELSE), Nanyang Technological University, 60 Nanyang Drive, Singapore 637551, Singapore; Singapore Center for Environmental Life Sciences Engineering (SCELSE), Nanyang Technological University, 60 Nanyang Drive, Singapore 637551, Singapore; Singapore Center for Environmental Life Sciences Engineering (SCELSE), Nanyang Technological University, 60 Nanyang Drive, Singapore 637551, Singapore; Singapore Center for Environmental Life Sciences Engineering (SCELSE), Nanyang Technological University, 60 Nanyang Drive, Singapore 637551, Singapore; Singapore Center for Environmental Life Sciences Engineering (SCELSE), Nanyang Technological University, 60 Nanyang Drive, Singapore 637551, Singapore; Singapore Center for Environmental Life Sciences Engineering (SCELSE), Nanyang Technological University, 60 Nanyang Drive, Singapore 637551, Singapore; Singapore Center for Environmental Life Sciences Engineering (SCELSE), Nanyang Technological University, 60 Nanyang Drive, Singapore 637551, Singapore; Singapore Center for Environmental Life Sciences Engineering (SCELSE), Nanyang Technological University, 60 Nanyang Drive, Singapore 637551, Singapore; Singapore Center for Environmental Life Sciences Engineering (SCELSE), Nanyang Technological University, 60 Nanyang Drive, Singapore 637551, Singapore; Singapore Center for Environmental Life Sciences Engineering (SCELSE), Nanyang Technological University, 60 Nanyang Drive, Singapore 637551, Singapore; Singapore Center for Environmental Life Sciences Engineering (SCELSE), Nanyang Technological University, 60 Nanyang Drive, Singapore 637551, Singapore; Singapore Center for Environmental Life Sciences Engineering (SCELSE), Nanyang Technological University, 60 Nanyang Drive, Singapore 637551, Singapore; Mandai Nature, 80 Mandai Lake Rd, Singapore 729826, Singapore; Animal Care Department, Mandai Wildlife Group, 80 Mandai Lake Rd, Singapore 729826, Singapore; Singapore Center for Environmental Life Sciences Engineering (SCELSE), Nanyang Technological University, 60 Nanyang Drive, Singapore 637551, Singapore; Singapore Center for Environmental Life Sciences Engineering (SCELSE), Nanyang Technological University, 60 Nanyang Drive, Singapore 637551, Singapore

**Keywords:** air microbiome, bioaerosols, microbial ecology, local and remote microbial sources, eDNA

## Abstract

Recent developments in aerobiology have enabled the investigation of airborne biomass with high temporal and taxonomic resolution. In this study, we assess the contributions of local sources to ambient air within a 160,000 m^2^ tropical avian park (AP). We sequenced and analyzed 120 air samples from seven locations situated 160 to 400 m apart, representing distinct microhabitats. Each microhabitat contained a characteristic air microbiome, defined by the abundance and richness of its airborne microbial community members, supported by both, PCoA and Random Forest analysis. Each outdoor microhabitat contained 1% to 18.6% location-specific taxa, while a core microbiome of 27.1% of the total taxa was shared. To identify and assess local sources, we compared the AP dataset with a DVE reference dataset from a location 2 km away, collected during a year-round sampling campaign. Intersection of data from the two sites demonstrated 61.6% of airborne species originated from local sources of the AP, 34.5% from ambient air background, and only 3.9% of species were specific to the DVE reference site. In-depth taxonomic analysis demonstrated association of bacteria-dominated air microbiomes with indoor spaces, while fungi-dominated airborne microbial biomass was predominant in outdoor settings with ample vegetation. The approach presented here demonstrates an ability to identify local source contributions against an ambient air background, despite the prevailing mixing of air masses caused by atmospheric turbulences.

Significance Statement This research describes qualitative and quantitative assessment of microbial contributions from local sources relative to the ambient air background. Longitudinal measurements of the ambient air background provide foundational data on the dynamics of airborne biomass. These provide a base for assessing deviations in microbial community composition resulting from rising atmospheric temperatures, particularly in urban settings. Identifying local source contributions will enable environmental forensics and surveillance of ambient air, thereby enabling the assessment of potential impacts on public health, agricultural production sites, as well as on terrestrial and aquatic ecosystems.

## Introduction

Over the past century, numerous contributions have been made to assess the occurrence and classification of atmospheric microorganisms and airborne biomass (1–3). Most of the early studies focused on the collection of fungal spores (4). In more recent decades, airborne biomass has been investigated based on smaller air volume samples collected over extended sampling durations (5, 6) or passively sampled dust particles as a means of aggregating airborne biomass (7–10). Due to limitations arising from low-biomass samples, in many instances targeted amplification-based techniques were used (11, 12), carrying the risks of sample contamination and amplification artefacts (13). Recent advances in air sampling techniques, however, effectively reduce sampling times from weeks/days to hours/minutes, while amassing sufficient airborne biomass to reliably conduct amplification-free comparative metagenomics analysis (13). The analytical approach of this study follows our previously developed protocol and pipeline based on high-volumetric air sampling, ultra-low biomass amassment, and deep metagenomics sequencing (13–16). The two core features of the pipeline are comparability between datasets (normalization), and standardization of the sensitivity for taxonomic identification of airborne microorganisms and other biological matter.

Our previous air microbiome studies (13–16) revealed the temporal and spatial dynamics of atmospheric microbial communities, particularly in a tropical setting (14). In addition, the recently observed phenomenon of the microbial community composition oscillating between day and night (diel cycle) has now been confirmed to also occur in temperate climates (15, 16). In particular, the vertical stratification of airborne microorganisms in the lower troposphere was demonstrated above and below the planetary boundary layer (16). Importantly, it was shown that atmospheric turbulences cause strong mixing of air masses during daytime below the boundary layer, resulting in largely homogeneous airborne microbial communities. This mechanism is particularly pronounced in the tropics, where upward convection is strong due to the high prevailing daytime temperatures. The resulting mixing of ambient air in a tropical urban setting demonstrated the absence of daytime vertical stratification on a 50-floor high-rise building (1 to 156 m) (16).

Using the above air sampling and analysis protocols, we assessed the contributions of microorganisms from local sources to ambient air across short distances (160 to 400 m) relative to an off-site urban reference (Day Variantion Experiments, DVE), located 2 km away. For our environmental sampling, we adopted the concept of microhabitats, defined as distinct airborne microbial communities within the scale of the investigated field site (400 m × 400 m). In this regard, we measured the contributions of local sources relative to the ambient air microbiome background of six outdoor sites and one indoor control site within an avian park (AP) in Singapore. We collected 36 m^3^ of air per sample, which contained the biomass from the ambient air background, as well as the specific contributions originating from each distinct microhabitat.

For the goals of this study, the following assumptions were made: (i) through a combination of atmospheric turbulence and horizontal transport of airmasses across a heterogeneous city surface that disrupts laminar air flow, the airmasses examined in our study were highly mixed and can, therefore, be considered to contain homogeneous airborne microbial communities; (ii) the timing of air sampling was chosen to occur at 1 h after sunrise in order to maximize the effects of daytime mixing at the sampling locations; (iii) for the above two reasons, we used an off-site reference dataset that averages data from four environmental timeseries across 1 year (2 h resolution). The experimental design chosen for this study identifies the contributions of local sources relative to the ambient air background within a daytime urban setting.

In summary, our data and analysis provide insights into how microhabitat characteristics impact qualitative and quantitative aspects of airborne microbial communities at the local scale, relative to the contributions from the core microbiome of the ambient air background.

## Results

**Fig. 1.**
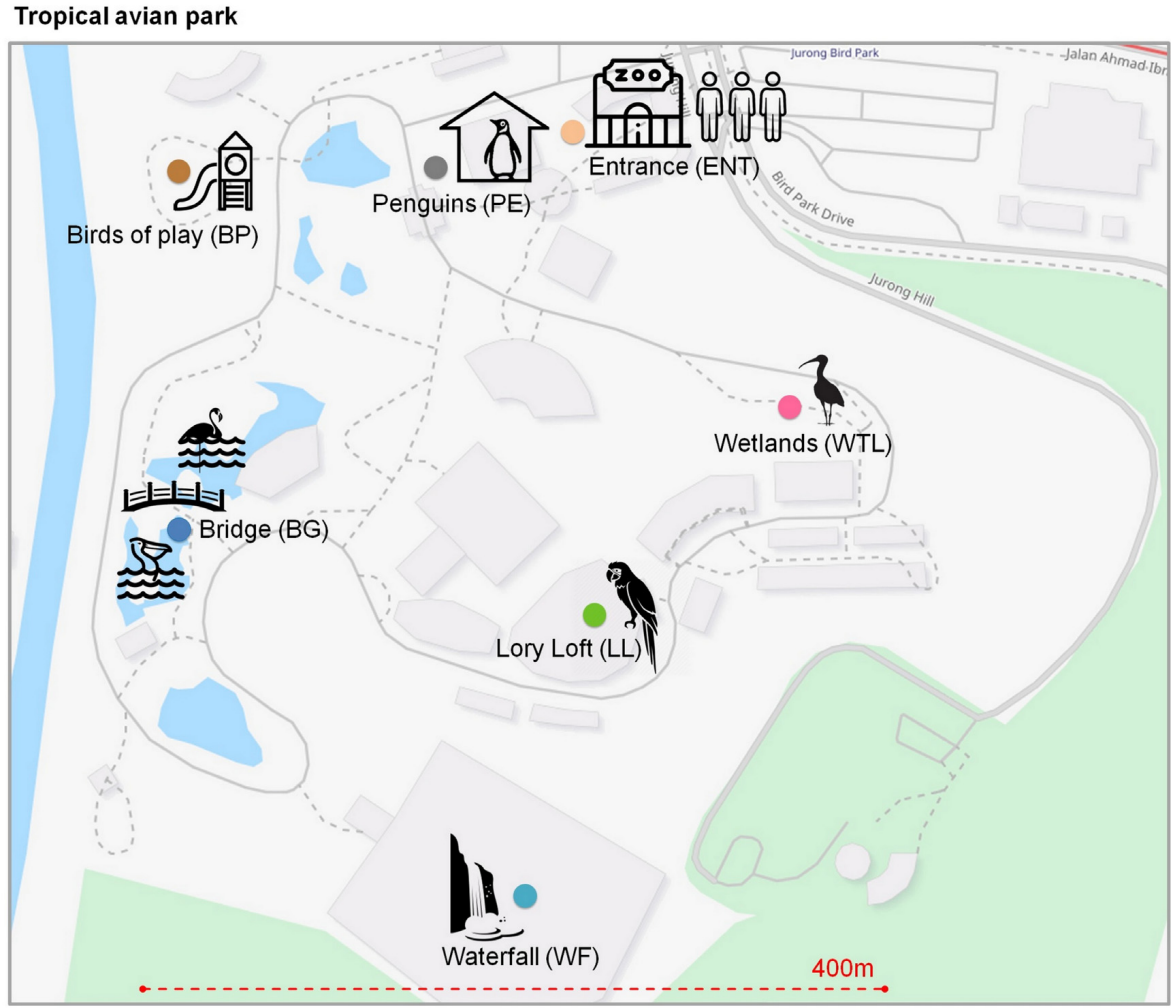
Experimental design and sampling at different microhabitat locations in the 160,000 m^2^ tropical AP. OpenStreetMap (www.openstreetmap.org) was used for the map representation.

A total of seven microhabitats comprising a total area of 160,000 m^2^ within a tropical AP (Jurong Bird Park, Singapore) were chosen for a multiday environmental survey (Fig. 1). The microhabitats represent sites with largely divergent local sources, such as plants, animals, and landscapes (e.g., lakes, ponds, and waterfalls). The locations are labeled as Entrance (high human occupancy, ENT), Wetlands (habitat for wetlands-dependent avian species, WTL), Lory Loft (high density avian host, LL), Waterfall (aerosolized fresh water, WF), Bridge (eutrophic water and animal hosts, BG), and Birds of Play (aqueous children's playground, BP). These six microhabitats represent outdoor locations that were contrasted by a single indoor mechanically ventilated location with high avian host occupancy (Penguin Enclosure, PE; Supplementary Material Appendix, Note S1).

Meteorological observations for the individual microhabitats of the AP are depicted in Supplementary Material Appendix, Figure S1A. The atmospheric temperature and relative humidity outdoors during sampling ranged from 25°C to 29°C and 67% to 95%, respectively. The highest humidity was detected at sites with aerosolized fresh water, e.g., waterfall (WF) and aqueous playground (BP) microhabitats. At the indoor penguin enclosure (PE), temperature and relative humidity were mechanically controlled, ranging from 13°C to 19°C, and 58% to 69%, respectively. Atmospheric CO_2_ concentrations outdoors ranged from 420 to 464 ppm, while indoor values at the PE ranged from 695 to 726 ppm. Outdoor wind speed ranged from 0.83 to 3.63 m s^−1^. Particle counts of the size 0.3 µm were detected in the air at maximal load both outdoors (0.17 to 4.44 × 10^6^) and indoors (0.16 to 1.49 × 10^6^; Supplementary Material Appendix, Figure S1B). Particles > 1 µm were 10x less common indoors, ranging from 341 to 0.13 × 10^6^, with outdoor values observed to be from 302 to 1.38 × 10^6^ (Supplementary Material Appendix, Figure S1C). Mild rain occurred during three of the six sampling periods (days 1, 4, and 6; Supplementary Material Appendix, Figure S1D).

**Fig. 2.**
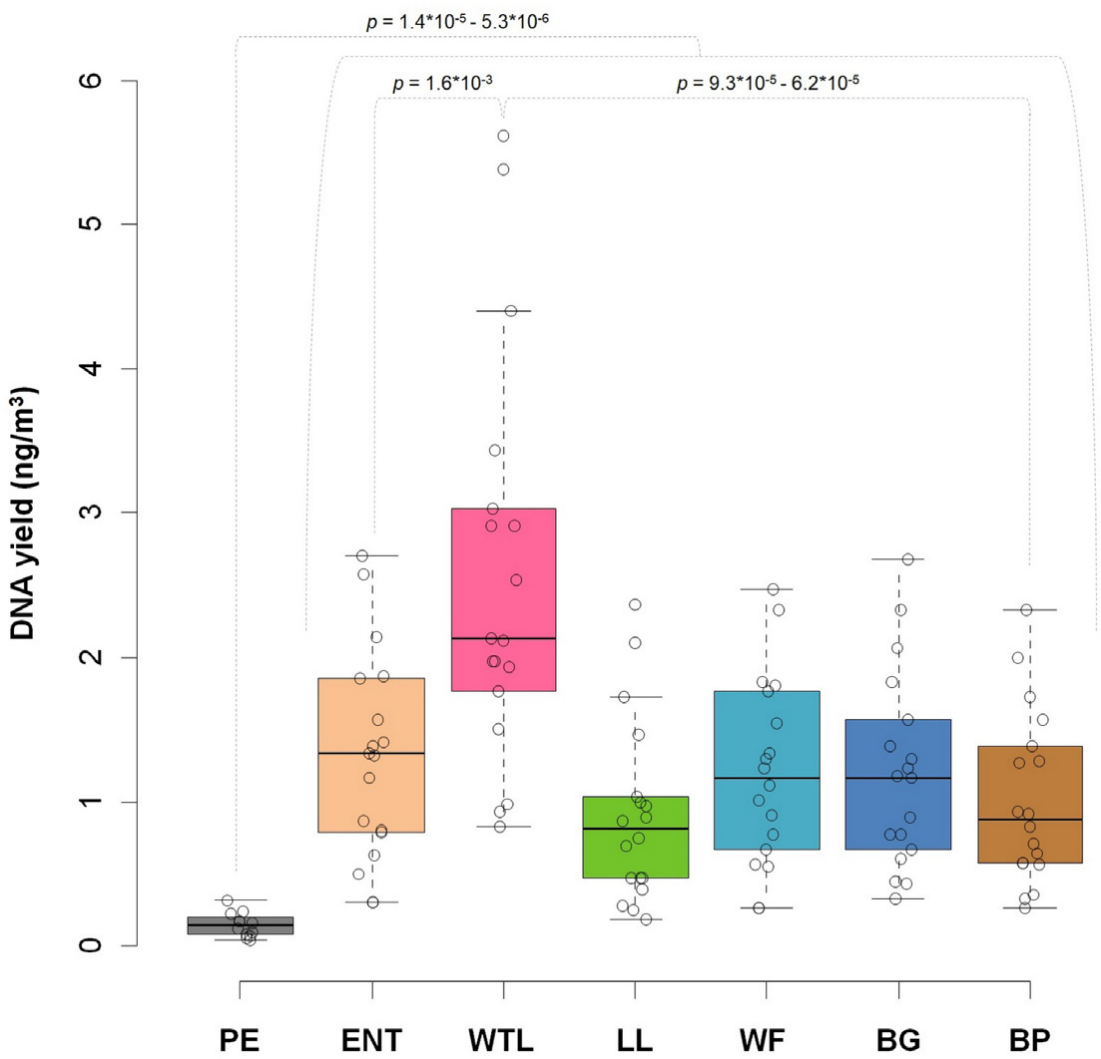
The total DNA yields (in ng m^−^^3^) isolated from the airborne biomass samples collected at distinct locations of the AP: PE—Penguin Enclosure, ENT—Entrance, WTL—Wetlands, LL—Lory Loft, WF—Waterfall, BG—Bridge, and BP—Birds of Play. *P*-values indicate significance of differences between microhabitat locations (assessed by Wilcoxon test).

In total, the analyzed dataset comprised 120 individual airborne biomass samples, from which DNA was extracted and quantified using a fluorometer. Observed yields varied up to 3.3-fold between outdoor locations (Fig. 2). The PE indoor location showed up to 8.9-fold less DNA concentration, compared to all outdoor locations (e.g., 10.8 to 22.7 ng and 30.0 to 202.2 ng for PE and WTL, respectively; Fig. 2).

**Fig. 3.**
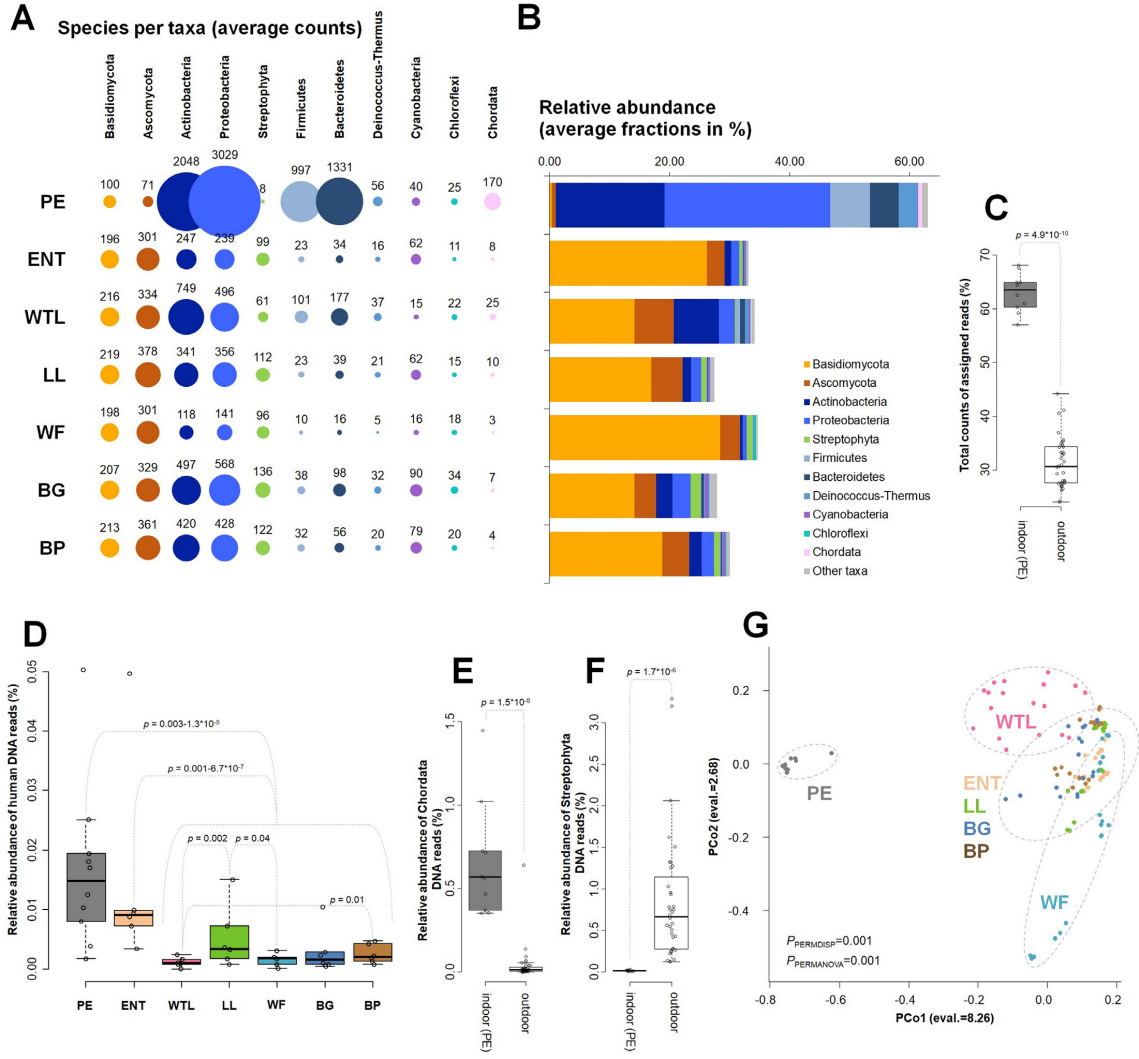
Composition and richness of the microbial communities across microhabitats of the AP. PE—Penguin Enclosure, ENT—Entrance, WTL—Wetlands, LL—Lory Loft, WF—Waterfall, BG—Bridge, and BP—Birds of Play. (A) Richness of the top 11 most abundant phyla across the microhabitats. Species counts are indicated. (B) Relative abundances (average fractions in %) of the top 11 most abundant phyla are plotted for different microhabitats. (C) Total counts of assigned reads for indoor and outdoor sampling at the microhabitats of the AP. *P*-value indicates significance of differences between indoor and outdoor microhabitats (assessed by Wilcoxon test). Relative abundance (in %) of human DNA (D), Chordata (E), and Streptophyta (F) in the environmental airborne samples across microhabitats of the AP. *P*-values indicate significance of the differences between microhabitat locations (assessed by Wilcoxon test). (G) Bray–Curtis dissimilarity distances between airborne samples of the microhabitats plotted in the first two principal coordinates. Significance of differences between clusters was assessed by PERMDISP and PERMANOVA tests.

**Fig. 4.**
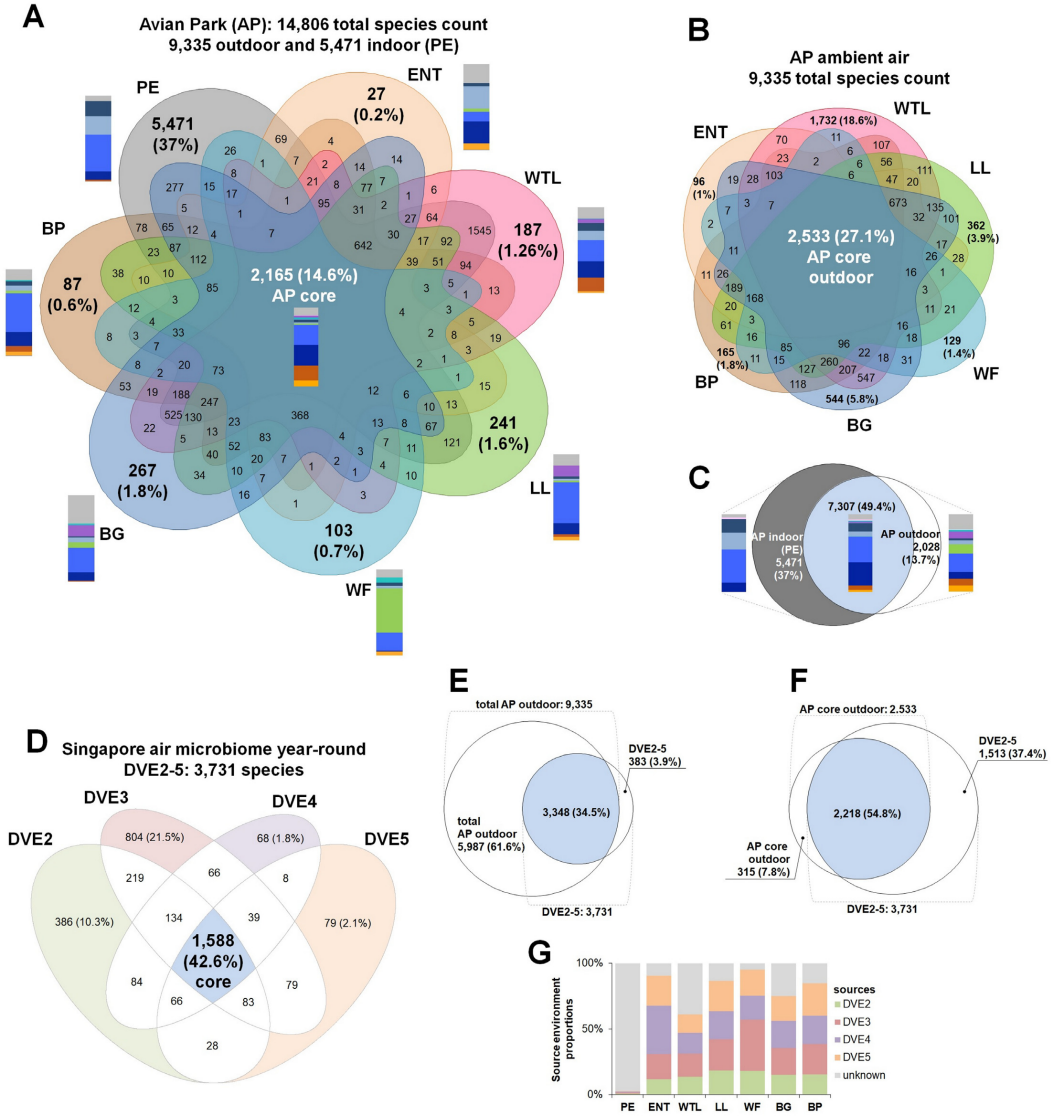
Local and remote sources for the air microbiome. Species-level analyses. PE—Penguin Enclosure, ENT—Entrance, WTL—Wetlands, LL—Lory Loft, WF—Waterfall, BG—Bridge, and BP—Birds of Play. (A) Venn-diagram of species co-occurring in seven microhabitats of the AP. The core species identifiable across all the microhabitats are indicated in white. Bars indicate the relative composition (in counts of species) of the corresponding location-specific microbial communities. (B) Species intersection between six outdoor locations. (C) Species intersection between the indoor (PE) location and all six outdoor locations. Bars indicate the relative composition (in counts of species) of the corresponding location-specific microbial communities. (D) Venn diagram of species co-occurring in four environmental time-series (Day Variation Experiments 2, 3, 4, and 5) conducted in Singapore throughout 2017 (14). Only time-matched 118 morning samples of the DVE2-5 collected during the time-intervals from 07:00 to 11:00 were considered. The core species identifiable across all time-series (DVE2-5) are highlighted in blue. (E) Intersection between the total AP outdoor species (9,335 species) and the Singapore air microbiome year-round (DVE2-5: 3,731 species). (F) Intersection between the AP core species (2,533 species) and the Singapore air microbiome year-round (DVE2-5: 3,731 species). (G) Microbial source tracking at the AP microhabitat locations. In the source tracking modeling, the DVE2-5 microbial communities were considered as sources while AP microhabitats were considered as sinks.

DNA sequencing libraries were prepared from the extracted DNA and sequenced on the Illumina HiSeq2500 platform to depths of 1.4 to 9.5 million reads per sample with a read length of 250 bp. All subsequent analyses were based on normalized datasets of ∼1.4 million reads for each sample (Figs 3 and 4). Following the previously established metagenomic analysis pipeline (13), we identified taxa at the phylum level, and displayed the top 11 groups (Fig. 3). This identified differences in richness (Fig. 3A) and abundance (Fig. 3B) of the airborne microbial communities at all sampling locations. In particular, the indoor setting (PE) was dominated by bacterial taxa (49% to 68%), with only 0.01% to 8% fungal and 0.35% to 1.45% Chordata (animal phylum, e.g., human, fish, and birds) DNA reads detected (Fig. 3B). In contrast, outdoor locations were dominated by Basidiomycota and Ascomycota fungi, with DNA read counts of 14% to 40% for fungi, only 1% to 21% for bacteria, and 0.0008% to 0.64% for Chordata (Fig. 3B). The identification rate in bacteria-dominated settings was substantially higher than for fungal-dominated settings, with 59% to 68% of the total reads being identifiable for the indoor microhabitat and 24% to 44% of the total reads for the six outdoor settings (Fig. 3C, Supplementary Material Appendix, Figure S2A).

Human genetic material was present at a significant level in the PE indoor microhabitat, as it is regularly accessed by the staff of the AP for feeding animals and cleaning the aviary (Fig. 3D). In outdoor microhabitats, human DNA reads were identified at a lesser degree, but were nevertheless detectable at all locations (Fig. 3D), demonstrating the sensitivity of the presented experimental approach. In addition to human DNA, the PE location was rich in other Chordata taxa (Fig. 3A and E), also encompassing DNA of penguin species (*Aptenodytes forsteri* and *Pygoscelis adeliae)*, as well as of various fish genera (e.g., *Oncorhynchus, Esox, Myripristis, Clupea, Salmo, Gadus*,and*Chanos*; Supplementary Material Appendix, Figure S3). In contrast, outdoor settings were enriched in members of the plant clade Streptophyta by a significant margin, compared to the indoor PE location (Fig. 3F).

Consequently, the PE microhabitat acts as an outgroup in the PCoA analysis (Fig. 3G) due to the characteristics described above, while clusters of all outdoor microhabitats largely overlap except for the WTL site. The WTL microhabitat was the most distinctive among all outdoor locations due to a larger fraction of bacteria detected (up to 21% of the microbial community; Fig. 3B). In particular, the increased abundance (Fig. 3A) and richness of Actinobacteria (Fig. 3B) is noteworthy.

Unsupervised classification of the air microbiomes by Random Forest (RF) analysis enabled differentiation between microbial communities from the seven tested microhabitats and attributed every sample to a particular location with high accuracy (RF error rate = 3.3%; Supplementary Material Appendix, Table S1). The uniquely identifiable structure of the microbial communities is indicative of the local dispersal of microorganisms that are released from nearby sources.

Based on the taxonomic classification of metagenomics data, the AP air microbiome comprises 14,806 taxa, of which 9,335 taxa were identified from outdoor sites, and 5,471 taxa being specific to the indoor PE location (Fig. 4A). This PE-specific microbiome was significantly more diverse than any of the outdoor-specific microbiomes that ranged from 27 (ENT) to 267 (BG) taxa (Fig. 4A). The core microbiome of the 120 air samples from the tropical AP constituted 2,165 taxa (Fig. 4A). The microbiome of the indoor PE site featured 12,778 species, of which 5,471 are PE-specific, while 9,335 species were sampled from all outdoor sites (Fig. 4B). About 37% of all species were specific to the PE microhabitat, 13.7% were specific to the outdoor air environment, and 49.4% were present in both environments (Fig. 4C). Importantly, a large segment of the outdoor-specific species comprised Streptophyta and fungal organisms, thereby contrasting the animal host-associated, bacteria-dominated indoor air microbiome.

Using year-round data from a nearby test site (Day Variation Experiments—DVE (14)), we assessed the ambient air microbial communities in proximity (2 km away from the AP), but with minimal human occupancy. These data consisted of time-matched samples (07:00 to 11:00), averaging 5-day time intervals during four sampling campaigns (DVE2-5) conducted 3 months apart, and can be considered as a year-round average of tropical ambient air. As an outcome, we determined the year-round air microbiome consists of 3,731 species, with a core microbiome of 1,588 species (Fig. 4D). The robustness of both environmental studies becomes apparent when assessing the intersection of the AP community richness (outdoors) and DVE2-5, with the AP airborne microbial community shares 34.5% of the total species count with the year-round time series samples from a different location (Fig. 4E). The shared airborne microbial taxa are even higher (54.8%) when the core communities of the AP outdoors are intersected with DVE2-5 (Fig. 4F). These 2,218 shared species, therefore, likely represent the microbial background that is typical for the tropical ambient air of this region. We also conducted source tracking analysis using SourceTracker2 (17) to assess the source environment proportions (DVE2-5) in sink samples from the microhabitat sites of the AP. The proportions of the source environment outdoors ranged from 61% (at the WTL site) to 95% (at the WF site; (Fig. 4G; Supplementary Material Appendix, Table S3). For the indoor PE location, the proportion of unknown source was 97% (Fig. 4G), which likely indicates a strong local source component.

## Discussion

Based on the results of our previous studies on temporal and spatial dynamics of airborne microbial communities, we hypothesize that contributions from local sources to ambient air can be qualitatively and quantitatively identified by high-volumetric air sampling and metagenomics analysis. Adopting the concept of atmospheric mixing (16), which results in a largely homogeneous ambient air background in the near-surface atmosphere, our experimental design investigated the air microbiomes of six outdoor locations and one indoor site at identical time points. Despite their proximity, these sites likely represent divergent microhabitats, due to their engineered landscaping and high occupancy rate of various avian species, as well as human visitors to the AP. We propose that the microhabitat-specific taxa identified for each location (Fig. 4) originated from local sources. It is important to note that the community composition for each site was shown to be robust, as our analysis is based on the two underlying principles of normalization of sequencing data to ∼1.4 million reads per sample, and statistical assessment of rare species contribution. Particularly, by conducting several rounds of random subsampling from the same dataset, we can show that 97% to 99% of the species are concordant in each of five subsamples (Supplementary Material Appendix, Figure S4). We, therefore, conclude that stochastic contributions to the microbial community structure are minimal, and the statistical analysis of our study is robust. This proceeding allows us to determine the contribution of specific local sources from each microhabitat relative to the ambient air background as defined above.

### Identification of the near-surface ambient air background

To define the ambient air background, we compared airborne microbial communities of 108 outdoor air samples (ENT, WTL, LL, WF, BG, and BP) to a reference dataset derived from an outdoor site of built environment 2 km away (DVE2-5, (14)). This reference site is characterized by sparse horticulture, the absence of animal hosts, as well as minimal human occupancy. For the purpose of this study, it is important to note that the airborne microbial community composition of the reference site remained largely unchanged within the sampling interval of 13 months (DVE2-5), despite opposing wind directions during the two monsoon seasons. This finding, therefore, supports the atmospheric mixing model (16) in an urban tropical setting and forms the basis for the ambient air background concept used for subsequent analysis of local source contributions.

### Location-specific contributions to the air microbiome

By intersecting both datasets (total AP outdoor and nearby test site DVE2-5, Fig. 4E), we show that 34.5% of airborne taxa are shared, e.g., forming the ambient air background, and 61.6% are specific to the six outdoor microhabitat sites. For the DVE2-5 reference site, only 3.9% of detected species were shown to be specific (Fig. 4E). Consequently, 89.7% of taxa of the reference dataset were contained within the AP microbiome, indicating minimal contributions of local sources to the air masses at the reference site.

The contributions from local sources to the air microbiomes of microhabitats ranged from 1% to 18.6% of total detected species, indicated by presence/absence analysis (Fig. 4B). Source tracking analysis (Fig. 4G), based on relative abundances of species in the airborne microbial community from AP outdoor sites and the urban reference site location (DVE), demonstrated that 61% to 95% of the air masses originated from the ambient air background (Fig. 4G). The remaining fraction of unknown sources ranged from 5% to 39%, being maximal for the WTL site (Fig. 4G). Thus, the contribution from the local microbial sources is more substantial at WTL, making this location distinct from the remaining five outdoor sites. Based on the results from our absence/presence and source tracking analyses, we would also like to note that despite the numerous species contributed by local sources (Fig. 4B), their relative abundance in the overall community structure appeared to be low (Fig. 4G).

Our metagenomics analysis of airborne biomass collected with high-volumetric air samplers expands on previous reports by enabling simultaneous observation of bacterial, fungal, plant, and vertebrate host taxa. Furthermore, the quantitative assessment of all identified taxa is presented on a unified scale. In contrast, previous reports largely relied on 16S sequencing for taxonomic identification, thereby ignoring the contributions from eukaryotic phyla. Nevertheless, both approaches, metagenomics and 16S amplicon sequencing, show that microhabitats generate a specific microbiome that reflects the contributions from local sources. In this regard, similar to the findings from our study, another study reported that bacteria from local sources comprised 50% to 61% of the bioaerosols in urban settings (18). Comparison of urban car parking lots to nature parks revealed that individual parks are characterized by unique bacterial signatures, while parking lots did not differ significantly from each other (19). Analysis of agricultural fields, suburban areas and forests indicated that the landscape topology has a greater impact on the composition of airborne bacteria than the meteorological characteristics at the sites (20). These previously published results support our starting hypothesis that contributions from local sources in different settings represent distinguishable microhabitats. The latter was further confirmed in our study by an unsupervised machine learning approach (RF analysis), providing accurate classification of samples across all microhabitats with an error rate of 3.3%.

### Outdoor vs. indoor air microbiomes

Local source contributions become particularly strong in the instance of our indoor test site (PE). This site is characterized by a high density of avian hosts and human occupancy, in combination with limited air exchange and air conditioning. The latter results in large ambient temperature differences, which are required for housing Antarctic bird species in a tropical environment. Due to the specific environmental settings at the PE indoor site, the airborne microbial communities are shifted from the fungal-dominated taxonomic profiles of the outdoor sites to a bacterial-dominated one. This strong deviation of the PE site from the air microbiome communities outdoors can be explained by several factors: (i) existence of specific microbial sources indoors, such as live feeding (fish) and avian feces, (ii) specific indoor microclimate with the ambient temperature being ∼15°C lower than outdoors, and most importantly (iii) high avian occupancy at the PE site with more than 30 individual penguins being present during air sampling. Overall, the largest contribution to the air microbiome is likely coming from the microorganisms associated with the keratin-rich plumage of penguins.

To identify the sources of the indoor microbiome, we performed source tracking analysis where the DVE2-5 reference outdoor samples were considered as the source. This source tracking modeling estimated the unknown fraction to be 97%, indicating that the PE indoor airborne biomass is generated mainly by local sources. The aviary hosted two bird species (*A. forsteri*and*P. adeliae*), which were accurately identified by our taxonomic analysis, underlining the sensitivity and resolution of our metagenomic analysis. In addition, aerosols generated by live feeding of the penguins with various fish of the genera *Oncorhynchus, Esox, Myripristis, Clupea, Salmo, Gadus*, and *Chanos* were also identified. The PE indoor air microbiome, drastically differing from microbiomes of the outdoor microhabitats and human skin microbiomes, suggests that the observed bacterial taxa are in fact associated with the avian host (Figs 3 and 4). Similarly, bacterial genera associated with the human skin microbiome were shown to be predominant in a study investigating indoor spaces within an urban subway network (21–23). Previously reported studies of human habitations did not find fungal taxa that were indicative of human presence (24), with the exception of the dandruff causing fungus Malassezia (25). Moreover, indoor fungal composition in the built-environment was shown to not be associated with building function or design, but rather dependent on the outdoor fungal composition of a particular climatic zone (26).

In our study, dominant bacterial species identified at the indoor PE site belong to the Actinobacteria and Proteobacteria phyla, as well as Firmicutes and Bacteroides. The remainder of the microbial diversity of the PE microhabitat can be explained by the fresh air intake, thus introducing the extant ambient air microbiome into the enclosure. Indeed, the richness of the PE microbial community is almost double that of the outdoor locations (Supplementary Material Appendix, Figure S2B).

Samples collected from the PE location showed the lowest DNA yields, despite the microbial community of this microhabitat being the richest (Fig. 3A). However, genomes of airborne bacteria are generally ∼10-times smaller compared to airborne fungal genomes (14). This difference in genome size likely explains the observed differences in extracted DNA yields between the bacteria-rich indoor PE microhabitat and the fungi-rich outdoor microhabitats.

In contrast to the PE location, all six outdoor microhabitats showed a high abundance and diversity of fungal taxa (Figs 3 and 4). In outdoor settings, a 7.6- to 17-fold increase of Streptophyta taxa was observed, while the abundance of Chordata taxa was reduced by 7.7- to 42.5-fold. These characteristics of outdoor and indoor locations likely reflect the evolutionary close links between fungi and plants, particularly wood-rotting saprophytes such as Basidiomycota, Ascomycota molds, and/or plant pathogens. The understanding of how host-specific microbiomes from animals and plants contribute to the ambient air microbiome, therefore, allows for the qualitative and quantitative assessment of local sources, even in outdoor settings. Despite the strong differences in airflow between outdoor and indoor settings, our study identified avian DNA in air samples taken in ambient air setting. Environmental DNA (eDNA) has previously been identified in bioaerosols in multiple studies (27, 28). However, as shown for the PE location, the capacity of our analytical pipeline to identify animal species in the form of eDNA is deemed minimal at larger distances from the source in outdoor settings, and likely to be successful only in closed quarters (29).

In addition to the host microbiomes from animals and plants, eutrophic water was identified as a strong local source, associated with increased abundances of Actinobacteria species (Fig. 3A and B). As the WTL setting contains large areas of surface water, which are being mechanically aerated, aerosols containing water-borne microorganisms are likely to be generated. Similarly, air samples from the WF microhabitat contained aerosolized biological matter, originating from water plants in a small river. However, the water quality of the WTL and WF sites largely differed due to the latter being sourced from a clean water supply.

### Conclusion

Our analyses indicate that microhabitats located within a small-scale area can be used to accurately identify contributions from local sources to the ambient air background. In-depth taxonomic analysis of indoor and outdoor microbiomes, and comparison to a reference dataset demonstrates strong differences in the airborne microbial community composition of indoor and outdoor settings, with animal and human host microbiomes being a major source of detected bacteria-dominated bioaerosols. An abundance of fungal taxa, frequently encountered in tropical settings, is associated with wood-rotting fungi belonging to the Basidiomycota phylum. The qualitative and quantitative assessment of local sources of airborne biomass against the ambient air background therefore allows for the identification of local source contributions even in naturally ventilated settings.

## Methods

### Sample collection

Airborne biomass samples were collected across six outdoor locations (ENT—Entrance, WTL—Wetlands, LL—Lory Loft, WF—Waterfall, BG—Bridge, and BP—Birds of Play) and one indoor location (PE) at the Jurong Bird Park, a tropical AP in Singapore (Fig. 1; Supplementary Material Appendix, Note S1). Outdoor samples were collected at the end of the Southwest Monsoon season in the period of 20 to 28 September.

Active collection of airborne biomass was performed using high-volumetric filter- and liquid-based samplers (13, 30, 31). To avoid the confounding effect of the diel cycle of airborne microbial communities (13, 14), all outdoor sampling was conducted synchronously across 6 days (08:00 to 10:00 local time) using 18 filter-based air samplers. Indoor sampling was conducted using both filter- and liquid-based samplers during seven nonconsecutive days. In total, 120 airborne biomass samples were collected.

The airborne biomass collection approach and subsequent sample processing have been described in detail in previous reports (13, 14). Briefly, all outdoor samples (108 samples) and some indoor samples (five samples; PE) were collected using SASS3100 high-volumetric filter-based air samplers (Research International, USA) with removable polystyrene filters (Part Number: 7100-134-232-01, 6 cm diameter, Research International) that allow for a high flow rate. Sampling was performed at 300 l min^−1^ air flowrate for 2 h. After sampling, the SASS filters were stored at −20°C until further processing. Three technical replicates were collected per outdoor location for every 2-h time interval of sampling.

A total of three filter blanks were collected as controls by installing a new filter on the air sampler on-site for about 5 s without running the sampler. The filter blanks were then analyzed using the same protocol as the air biomass samples. Summary statistics for the metagenomic analysis of the filter blank samples are provided in Supplementary Material Appendix, Table S2.

Within the indoor PE, the remaining seven samples were collected using Coriolis Micro liquid-based air samplers (Bertin Instruments, France). The Coriolis Micro collected 54 m^3^ of air (3 h at 300 l min^−1^) in 15 ml of double-distilled water in a sterilized cone. Concordance between the SASS and Coriolis Micro metagenomic sequencing approaches is depicted in Supplementary Material Appendix, Figure S5 (for more information refer to (30)).

To compare the AP microbiome with the regional tropical microbiome, we also used reference data of the year-round Singapore DVE previously reported (14). In this study, we conducted four sampling campaigns, each 3 months apart (DVE2-DVE5), at an urban test location with minimal human occupancy. For each sampling campaign (DVE2-5), samples were collected with the same instrumentation used in the current study (SASS3100 samplers) during 12 time-intervals (2 h each) for five consecutive days. Identical sampling protocols were used for the collection of airborne biomass at the urban site and the outdoor sites of the AP. To compare the year-round sampling data (DVE2-5) with the AP data, we selected samples collected at corresponding time intervals, i.e., from 07:00 to 11:00 AM, to avoid confounding the comparison with diel fluctuations in community composition.

### Sample filtration

Technical replicates were processed separately. The SASS removable filters and the Coriolis Micro liquid samples were both filtered on an Anodisc inorganic filter membrane (Cytiva, USA). Each SASS filter was placed in a 5 ml sterile tube with 2 ml PBS and 0.1% Triton X-100 (PBS-T). The filters were squeezed with sterile forceps to distribute the buffer to all fibers. The tube containing the soaked filter was then placed in a sonication bath for 1 min. After sonication, the liquid was poured into an open 10 ml syringe that had been inserted into a 50 ml sterile Falcon Tube. The steps starting from emersion of the filter in 2 ml of PBS-T were repeated once. The SASS filter was then transferred to the 10 ml syringe in the 50 ml falcon tube and centrifuged for 5 min at 5,000 × *g* (RT). The wash steps were then repeated, using fresh 2 ml of PBS-T for a total of three washes. The combined liquid from the three washes was then filtered onto an Anodisc and placed into a 5 ml MoBio bead tube for DNA extraction.

### DNA extraction

DNeasy PowerWater DNA Isolation Kit (Qiagen, Germany) protocol was followed for DNA extraction. Briefly, 1 ml PW1 and 10 μl Proteinase K (20 μl ml^−1^ final concentration) were added to the bead tube containing the Anodisc and incubated overnight at 55°C. The tube was then vortexed for 3 min, followed by sonication in an ultrasonic bath for 30 min at 65°C in sweep mode before vortexing for another 5 min. The supernatant was then used for DNA extraction, following the manufacturer's protocol. The DNA was eluted from the column using 60 μl of PW6. The eluate was then reloaded onto the spin column for a second time to maximize DNA recovery. Extracted DNA samples were quantitated on a Qubit 2.0 fluorometer, using the Qubit dsDNA HS (High Sensitivity) Assay Kit (Invitrogen, USA).

### Metagenomics sequencing

Immediately prior to library preparation, sample quantitation was repeated on a Promega QuantiFluor fluorometer, using Invitrogen's Picogreen assay. If the concentration of a sample determined by Qubit and Picogreen varied by more than 10%, quantitation was repeated for a third time using the Picogreen assay.

Next-generation sequencing libraries were prepared manually with the Swift Biosciences’ Accel-NGS 2S Plus DNA kit, following the manufacturer's instructions. For samples with a concentration of > 0.25 ng μl^−1^, the starting amount of DNA for library preparation was normalized to 5 ng. DNA shearing was performed on either a Covaris S220 or E220 focused-ultrasonicator with the following settings: Peak Power: 175, Duty Factor: 5.0, Cycles/Burst: 200, and Run Time: 90 s. All libraries were dual-barcoded, using Swift Biosciences’ 2S Dual Indexing kit. PCR amplification selectively enriched for library fragments using adapters ligated on both ends of the DNA strands. The PCR cycles were normalized to eight for all libraries with a starting amount of 4 to 5 ng of DNA. For samples with less than 4 ng of DNA, amplification cycles were adjusted as follows: 3.0 to 3.9 ng: 9 cycles, 2.0 to 2.9 ng: 11 cycles, 1.0 to 1.9 ng: 13 cycles, and < 1 ng: 15 cycles. Size-selection was omitted for all libraries. All blanks were amplified with eight PCR cycles.

  Library quantitation was performed using Invitrogen's Picogreen assay and the average library size was determined using a Bioanalyzer DNA 7500 chip (Agilent, USA). Library concentrations were normalized to 4 nM and the concentration was validated by qPCR on a ViiA-7 real-time thermocycler (Applied Biosystems, USA), using Kapa Biosystem's Library Quantification Kit for Illumina sequencing platforms. For blanks, the stock libraries were used for qPCR. Libraries were then pooled at equal volumes and sequenced on Illumina HiSeq2500 Rapid runs at a final concentration of 10 to 12 pM and a read-length of 250 bp paired-end (Illumina V2 Rapid sequencing reagents).

### High-throughput sequencing data processing and analysis

Metagenomics data generated for the air samples were processed for adaptor removal and quality trimming with a Phred quality score threshold of Q20 (-q 20) and with a minimum read-length of 30 bp (–minimum-length 30) using Cutadapt v. 1.8.1 (32). The trimmed reads were then normalized to the smallest data size per sample (1,384,618 million reads) by random selection of reads from each sample. The normalized data were aligned against the NCBI nonredundant (NR) protein database (downloaded on 22.11.2019) using the sensitive taxonomic classification tool Kaiju v.1.7.2 (33). Resulting alignments were imported into MEGAN v.6.18, which assigns taxons based on NCBI taxonomy (34). To achieve the desired taxonomic specificity, we used the following filtering parameters: minScore = 100, minComplexity = 0.33, minSupport = 25, maxExpected = 0.01, minSupportPercent = 0, lcaPercent  = 100, paired = false, and topPercentage = 100. Lowest Common Ancestor (LCA) for each read in the NCBI taxonomy is assigned using MEGAN's LCA algorithm.

The reference data of the DVE sampling survey were reanalyzed using the same Kaiju protocols and NR protein database as described in this section above, to ensure comparability with the metagenomic data obtained from the AP.

### Statistical analysis

The Wilcoxon test (35) was used to assess significance of differences between the indoor (PE) and outdoor microhabitat locations as implemented in R v.4.0.2. Bray–Curtis dissimilarity distances among centroids for each sample series were calculated in *vegan* package in R v.4.0.2 (36) to visualize multivariate patterns in microbial communities. Permutation test for homogeneity of multivariate dispersions (PERMDISP) (37) and analysis of variance (PERMANOVA) (38) were used to assess significance of differences between the clusters of samples as implemented in vegan package (32) in R v.4.0.2. Principal Coordinates (PCo) were used as an ordination method (39). Alfa diversity index *chao1* was calculated in R v.4.0.2. RF analysis was performed using the *caret* package v.6.0-86 (40) in R v.4.0.2. The number of trees in the RF was specified using flag ntree = 501, type of RF was set to “classification”. Venn-diagrams were created using *eulerr* v.6.1.1 (41) and *ggVennDiagram* packages in R v.4.0.2. Source environment proportions were estimated using SourceTracker2 (17) and 40 independent observations from each source environment (DVE2-5). Technical replicates were averaged per time point. In total, 36 independent observations outdoors and 10 indoors were considered as sinks.

### Collection of sensor data

Sensor data were captured locally using Met ONE HPPC 6+ (Beckman Coulter, USA) to measure particle counts (size range: 0.3, 0.5, 1.0, 2.0, 5.0, and 10 μm), CO_2_ sensor—CP11 (Rotronic, Switzerland) for CO_2_ (ppm), and VelociCalc Air Velocity Meter 9545 (TSI, USA) for wind-speed (m s^−1^), temperature (°C), and relative humidity (%). All sensors were placed near the air samplers (0.3 to 3 m away) under a roof to protect them from direct rain and sunlight. Sensor instruments were set to collect data at 1-min intervals. The collected data were then averaged for the 2 h sampling-time intervals. Meteorological conditions across different microhabitats are summarized in Supplementary Material Appendix, Figure S1.

## Supplementary Material

pgac043_Supplemental_File

## Data Availability

The raw metagenomic sequencing data reported in this paper have been deposited in the National Center for Biotechnology Information (NCBI) database (BioProject ID code PRJNA775663).

## References

[1] Pasteur LA . 1861. On the doctrine of spontaneous generation. Ann Sci Nat Zool. 16:5–98.

[2] Fröhlich-Nowoisky J , et al. 2016. Bioaerosols in the Earth system: climate, health, and ecosystem interactions. Atmos Res. 182:346–376.

[3] Kim KH , Kabir E, Jahan SA. 2018. Airborne bioaerosols and their impact on human health. J Environ Sci. 67:23–35. 10.1016/j.jes.2017.08.027 PMC7128579 29778157

[4] Gregory PH . 1952. Spore content of the atmosphrere near the ground. Nature. 170:475–477. 12993203 10.1038/170475a0

[5] Franzetti A , Gandolfi I, Gaspari E, Ambrosini R, Bestetti G. 2011. Seasonal variability of bacteria in fine and coarse urban air particulate matter. Appl Microbiol Biotechnol. 90:745–753. 21184061 10.1007/s00253-010-3048-7

[6] Bowers RM , et al. 2013. Seasonal variability in bacterial and fungal diversity of the near-surface atmosphere. Environ Sci Technol. 47:12097–12106. 24083487 10.1021/es402970s

[7] Adams RI , et al. 2015. Passive dust collectors for assessing airborne microbial material. Microbiome. 3:46. 26434807 10.1186/s40168-015-0112-7 PMC4593205

[8] Kellogg CA , Griffin DW. 2006. Aerobiology and the global transport of desert dust. Trends Ecol Evol. 21:638–644. 16843565 10.1016/j.tree.2006.07.004

[9] Barberan A , Henley J, Fierer N, Casamayor EO. 2014. Structure, inter-annual recurrence, and global-scale connectivity of airborne microbial communities. Sci Total Environ. 487:187–195. 24784743 10.1016/j.scitotenv.2014.04.030

[10] Woo C , An C, Xu S, Yi SM, Yamamoto N. 2018. Taxonomic diversity of fungi deposited from the atmosphere. ISME J. 12:2051–2060. 29849168 10.1038/s41396-018-0160-7 PMC6051994

[11] Yooseph S. et al. 2013. A metagenomic framework for the study of airborne microbial communities. PLoS ONE. 8:e81862. 24349140 10.1371/journal.pone.0081862 PMC3859506

[12] Yamamoto N , et al. 2012. Particle-size distributions and seasonal diversity of allergenic and pathogenic fungi in outdoor air. ISME J. 6:1801–1811. 22476354 10.1038/ismej.2012.30 PMC3446800

[13] Luhung I , et al. 2021. Experimental parameters defining ultra-low biomass bioaerosol analysis. npj Biofilms Microbiomes. 7:37. 33863892 10.1038/s41522-021-00209-4 PMC8052325

[14] Gusareva ES , et al. 2019. Microbial communities in the tropical air ecosystem follow a precise diel cycle. Proc Natl Acad Sci. 116:23299–23308. 31659049 10.1073/pnas.1908493116 PMC6859341

[15] Gusareva ES , et al. 2020. Taxonomic composition and seasonal dynamics of the air microbiome in West Siberia. Sci Rep. 10:21515. 33299064 10.1038/s41598-020-78604-8 PMC7726148

[16] Drautz-Moses DI , et al. 2022. Vertical stratification of the air microbiome in the lower troposphere. Proc Natl Acad Sci. 119:e2117293119. 35131944 10.1073/pnas.2117293119 PMC8851546

[17] Knights D , et al. 2011. Bayesian community-wide culture-independent microbial source tracking. Nat Methods. 8:761–763. 21765408 10.1038/nmeth.1650 PMC3791591

[18] Dueker ME , French S, O'Mullan GD. 2018. Comparison of bacterial diversity in air and water of a major urban center. Front Microbiol. 9:2868. 30555433 10.3389/fmicb.2018.02868 PMC6282627

[19] Mhuireach G , et al. 2016. Urban greenness influences airborne bacterial community composition. Sci Total Environ. 571:680–687. 27418518 10.1016/j.scitotenv.2016.07.037

[20] Bowers RM , McLetchie S, Knight R, Fierer N. 2011. Spatial variability in airborne bacterial communities across land-use types and their relationship to the bacterial communities of potential source environments. ISME J. 5:601–612. 21048802 10.1038/ismej.2010.167 PMC3105744

[21] Leung MH , Wilkins D, Li EK, Kong FK, Lee PK. 2014. Indoor-air microbiome in an urban subway network: diversity and dynamics. Appl Environ Microbiol. 80:6760–6770. 25172855 10.1128/AEM.02244-14 PMC4249038

[22] Leung MHY , et al. 2021. Characterization of the public transit air microbiome and resistome reveals geographical specificity. Microbiome. 9:112. 34039416 10.1186/s40168-021-01044-7 PMC8157753

[23] Robertson CE , et al. 2013. Culture-independent analysis of aerosol microbiology in a metropolitan subway system. Appl Environ Microbiol. 79:3485–3493. 23542619 10.1128/AEM.00331-13 PMC3648054

[24] Adams RI , Miletto M, Taylor JW, Bruns TD. 2013. Dispersal in microbes: fungi in indoor air are dominated by outdoor air and show dispersal limitation at short distances. ISME J. 7:1262–1273. 23426013 10.1038/ismej.2013.28 PMC3695294

[25] Rudramurthy SM , et al. 2014. Association of Malassezia species with dandruff. Ind J Med Res. 139:431–437. PMC4069738 24820838

[26] Amend AS , Seifert KA, Samson R, Bruns TD. 2010. Indoor fungal composition is geographically patterned and more diverse in temperate zones than in the tropics. Proc Natl Acad Sci. 107:13748–13753. 20616017 10.1073/pnas.1000454107 PMC2922287

[27] Grzyb J , Lenart-Boroń A. 2019. Bacterial bioaerosol concentration and size distribution in the selected animal premises in a zoological garden. Aerobiologia. 35:253–268.

[28] Lynggaard C , et al. 2021. Airborne environmental DNA for terrestrial vertebrate community monitoring. Curr Biol. 32:701–707.e5. 10.1016/j.cub.2021.12.014 PMC8837273 34995490

[29] Clare EL , et al. 2021. eDNAir: proof of concept that animal DNA can be collected from air sampling. PeerJ. 9:e11030. 33850648 10.7717/peerj.11030 PMC8019316

[30] Dybwad M , Skogan G, Blatny JM. 2014. Comparative testing and evaluation of nine different air samplers: end-to-end sampling efficiencies as specific performance measurements for bioaerosol applications. Aerosol Sci Technol. 48:282–295.

[31] Els N , et al. 2019. Microbial composition in seasonal time series of free tropospheric air and precipitation reveals community separation. Aerobiologia. 35:671–701.

[32] Martin M , 2011. Cutadapt removes adapter sequences from high-throughput sequencing reads. EMBnet J. 17:10–12.

[33] Menzel P , Ng KL, Krogh A. 2016. Fast and sensitive taxonomic classification for metagenomics with Kaiju. Nat Commun. 7:11257. 27071849 10.1038/ncomms11257 PMC4833860

[34] Huson DH , et al. 2016. MEGAN community edition - interactive exploration and analysis of large-scale microbiome sequencing data. PLoS Comput Biol. 12:e1004957. 27327495 10.1371/journal.pcbi.1004957 PMC4915700

[35] Bauer DF . 1972. Constructing confidence sets using rank statistics. J Am Stat Assoc. 67:687–690.

[36] RCoreTeam . 2017. R: A Language and Environment for Statistical Computing (R Foundation for Statistical Computing). Vienna.

[37] Anderson MJ . 2006. Distance-based tests for homogeneity of multivariate dispersions. Biometrics. 62:245–253. 16542252 10.1111/j.1541-0420.2005.00440.x

[38] Anderson MJ . 2008. A new method for non-parametric multivariate analysis of variance. Austral Ecol. 26:32–46.

[39] Gower JC . 1966. Some distance properties of latent root and vector methods used in multivariate analysis. Biometrika. 53:325–338.

[40] Kuhn M . 2008. Building predictive models in R using the caret package. J Stat Softw. 28:1–26.27774042

[41] Larsson J , Gustafsson P. 2018. A case study in fitting area-proportional Euler diagrams with ellipses using Eulerr. Proceedings of International Workshop on Set Visualization and Reasoning. 2116:84–91.. Edinburgh.

