# Supplementary material for: Short-range contributions of local sources to ambient air
Source: PNAS Nexus. 2022 Apr 14;1(2):pgac043. doi: 10.1093/pnasnexus/pgac043 (PMC9802476; doi:10.1093/pnasnexus/pgac043)
Supplement: pgac043_Supplemental_File [file pgac043_supplemental_file.docx]

# Supplementary information

**Short range contributions of local sources to ambient air**

Elena S. Gusareva^1,2*^, Nicolas E. Gaultier^1*^, Akira Uchida^1^, Balakrishnan N. V. Premkrishnan^1^, Cassie E. Heinle^1^, Wen Jia Phung^1^, Anthony Wong^1^, Kenny J.X. Lau^1^, Zhei Hwee Yap^1^, Yanqing Koh^1^, Poh Nee Ang^1^, Alexander Putra^1^, Deepa Panicker^1^, Jessica Grace Huiyi Lee^3^, Luis Carlos Neves^4^, Daniela I. Drautz-Moses^1^, and Stephan C. Schuster^1^.

^1^Singapore Center for Environmental Life Sciences Engineering (SCELSE), Nanyang Technological University, 60 Nanyang Drive, 637551 Singapore.

^2^The Asian School of the Environment, Nanyang Technological University, 62 Nanyang Drive, 637459 Singapore.

^3^Mandai Nature, 80 Mandai Lake Rd, 729826 Singapore.

^4^Animal Care Department, Mandai Wildlife Group, 80 Mandai Lake Rd, 729826 Singapore.

*These authors contributed equally to this work

To whom correspondence should be addressed. Elena S. Gusareva [egusareva@ntu.edu.sg](mailto:egusareva@ntu.edu.sg), and Stephan C. Schuster, [SCSchuster@ntu.edu.sg](mailto:SCSchuster@ntu.edu.sg), [stephan.c.schuster@gmail.com](mailto:stephan.c.schuster@gmail.com)

**Supplementary Note S1.**

**Microhabitats of the avian park**

The avian tropical park (Jurong Bird Park, Singapore) is a 21 ha hillside natural park housing approximately 4,300 birds across close to 450 species.

**Entrance** area of the avian park includes ticket offices, ticket checkpoint, gift shops and restaurants. The avian park annual capacity is more than 850,000 visitors (pre-pandemic). Air sampling was conducted near food courts and ticket offices.

**Lory Loft** is a large 3,000 m^2^ walk-in aviary with the ambience of a rainforest valley. At the time of the experiment, it housed around 200 free-flying birds of 15 species from the Australasian region, including lories, lorikeets, and other species such as eclectus parrots, king parrots, and cockatoos. Air sampling was conducted inside the aviary where birds interact with visitors.

**Waterfall** is a large 20,000 m^2^ open-air walk-in aviary with a height of 35 m and houses a 30 m man-made waterfall. The environment in the aviary is a typical tropical rainforest. It houses hundreds of free-flying birds from 50 African species and 10,000 plants with 125 species of trees, bamboo, palms and ground-cover vegetation, including various species of birds around the world such as guineafowl, crowned pigeons, roseate spoonbill, a variety of starling species and many more. Air sampling was conducted at the highest point accessible near the cascade.

**Wetlands** is a zone that comprises two large enclosures that recreates the natural wetlands environment to provide habitat for wetlands-dependent species (scarlet ibis, night herons, stork, cormorants, etc.). Air sampling was conducted on the visitors’ pathway outside and adjacent to the enclosures and a small pond.

**Bridge** area is between a shallow water lake with an island where pelicans create communal nests, and a flamingo pool. Some of the species here include great white pelicans, Australian and Dalmatian pelicans, as well as greater flamingos. Air sampling was conducted on a bridge in-between the pelican and flamingo lakes.

**Birds of Play** area of the avian park includes a water playground for children, restaurants, and a gift shop. The playground was empty during the air sampling.

**Penguin Enclosure** is a 1,630 m^2^ indoor exhibit with the temperature kept at ~10-15^o^C and a special lighting system. The interior of penguin enclosure is constructed from timber beams and wooden flooring. The aviary is fenced off by a glass wall from the area where the visitors are located. Over 30 penguins were kept in this zone during air sampling. The penguins included the king and Humboldt penguins, but also silver and gray-hooded gulls.


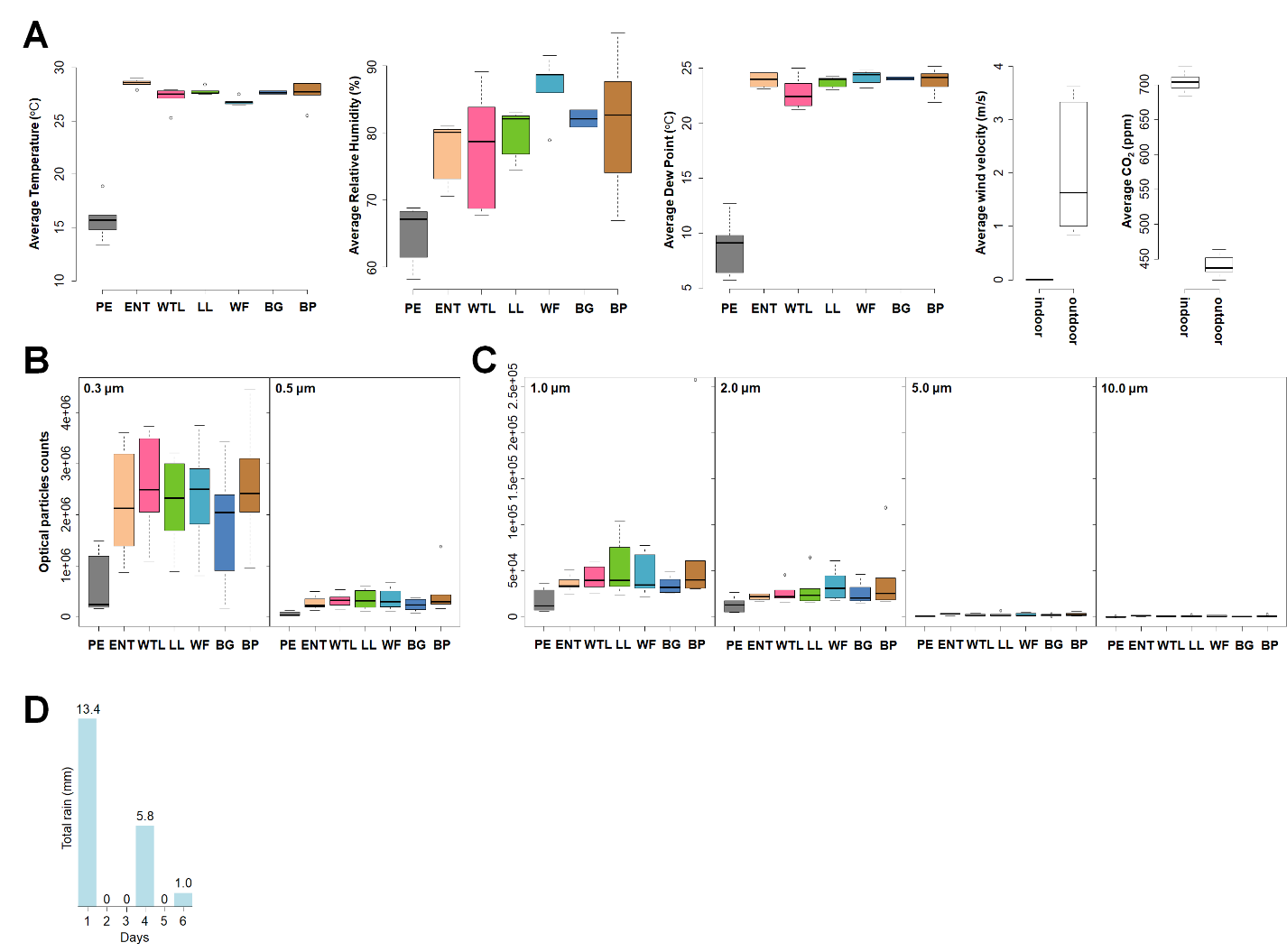


**Supplementary Fig. S1.** Meteorological observations for the individual microhabitats of the avian park during the sampling campaign. PE – Penguin Enclosure, ENT – Entrance, WTL – Wetlands, LL – Lory Loft, WF – Waterfall, BG – Bridge, BP – Birds of Play. **A.** Meteorological characteristics: atmospheric temperature, relative humidity, dew point values, wing velocity, and atmospheric carbon dioxide (CO_2_). Temperature, relative humidity, dew point, air carbon dioxide (CO_2_), wind velocity, and optical particle counts were collected every 1 min. The collected data were then averaged for the 2 h sampling time-intervals. Collectable optical particle counts of size 0.3 µm and 0.5 µm (**B**) and size > 1 µm (**C**) at different microhabitats of the avian park. **D.** Total rain during 2 h sampling intervals across 6 days of sampling.


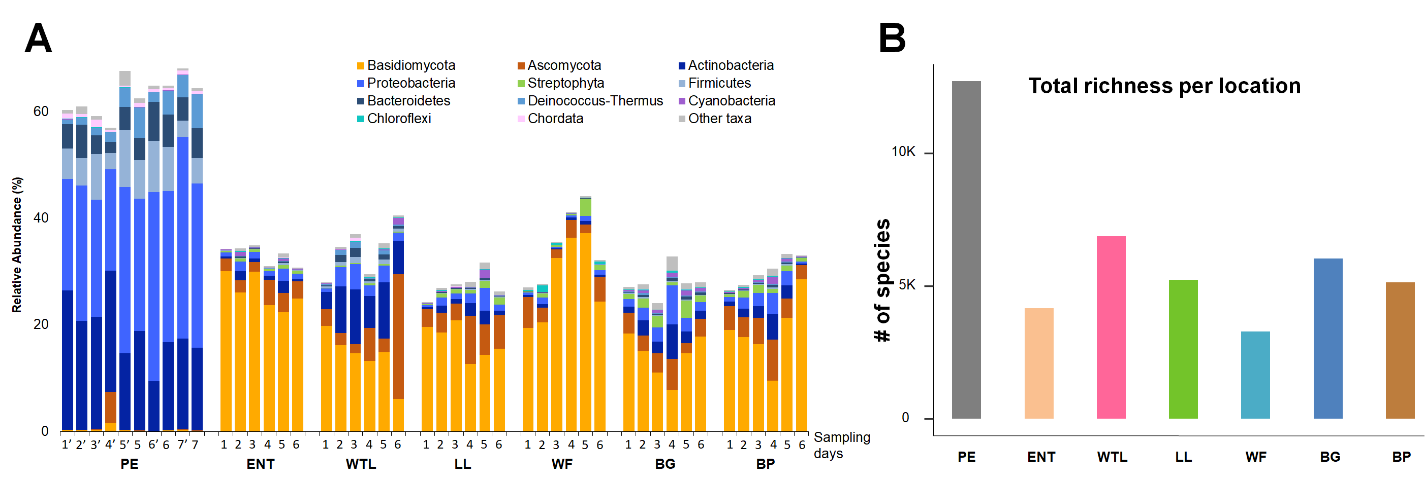


**Supplementary Fig. S2.** Abundance and richness of microbial communities at different microhabitats. **A.** Relative abundances (in %) of the top-10 most abundant phyla are plotted for different microhabitats (PE – Penguin Enclosure, ENT – Entrance, WTL – Wetlands, LL – Lory Loft, WF – Waterfall, BG – Bridge, BP – Birds of Play). Samples were collected in triplicates (with exception for PE location), technical replicates were averaged. Samples indicated with (‘) were collected using Coriolis Micro (Bertin Instruments, France), other samples were collected using SASS3100 (Research International, USA) air sampling techniques. **B.** Total richness of microbial communities at different sites.

Of the total genetic material analyzed, DNA reads assigned to taxa ranged between 59-68% for samples collected in the indoor PE microhabitat, and 24-44% for samples collected outdoors. This can be explained the bias of databases towards bacterial species, in the absence of sufficient genomic information for in-depth taxonomic classification of eukaryotes, particularly fungi and plants. Thus, due to limitations of existing public sequence databases, some sequencing reads do not result in meaningful alignments and are assigned to the “no-hits” category. The unassigned reads category includes low-complexity, repetitive DNA sequences or multiple alignments beyond domain-level.

**A**

**
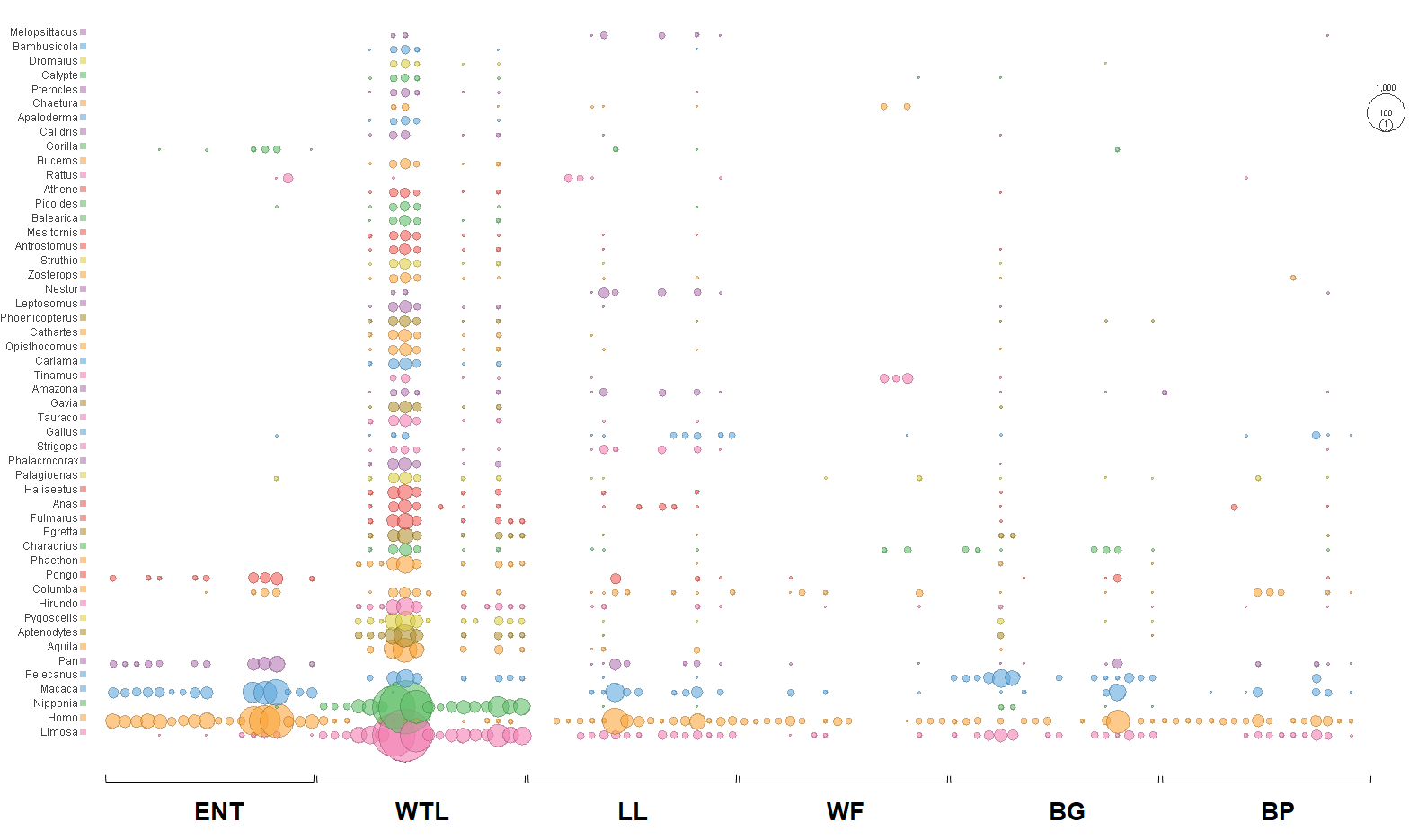
**

**B**

**
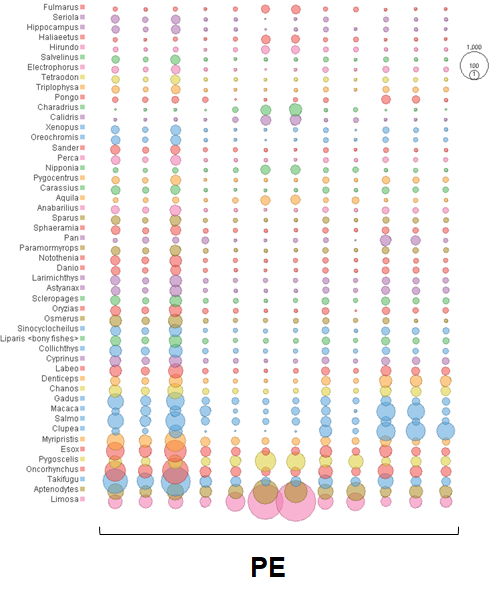
**

**Supplementary Fig. S3.** The top 50 Chordata genera identified outdoors (ENT – Entrance, WTL – Wetlands, LL – Lory Loft, WF – Waterfall, BG – Bridge, BP – Birds of Play) and indoors (PE – Penguin Enclosure) at the tropical avian park.


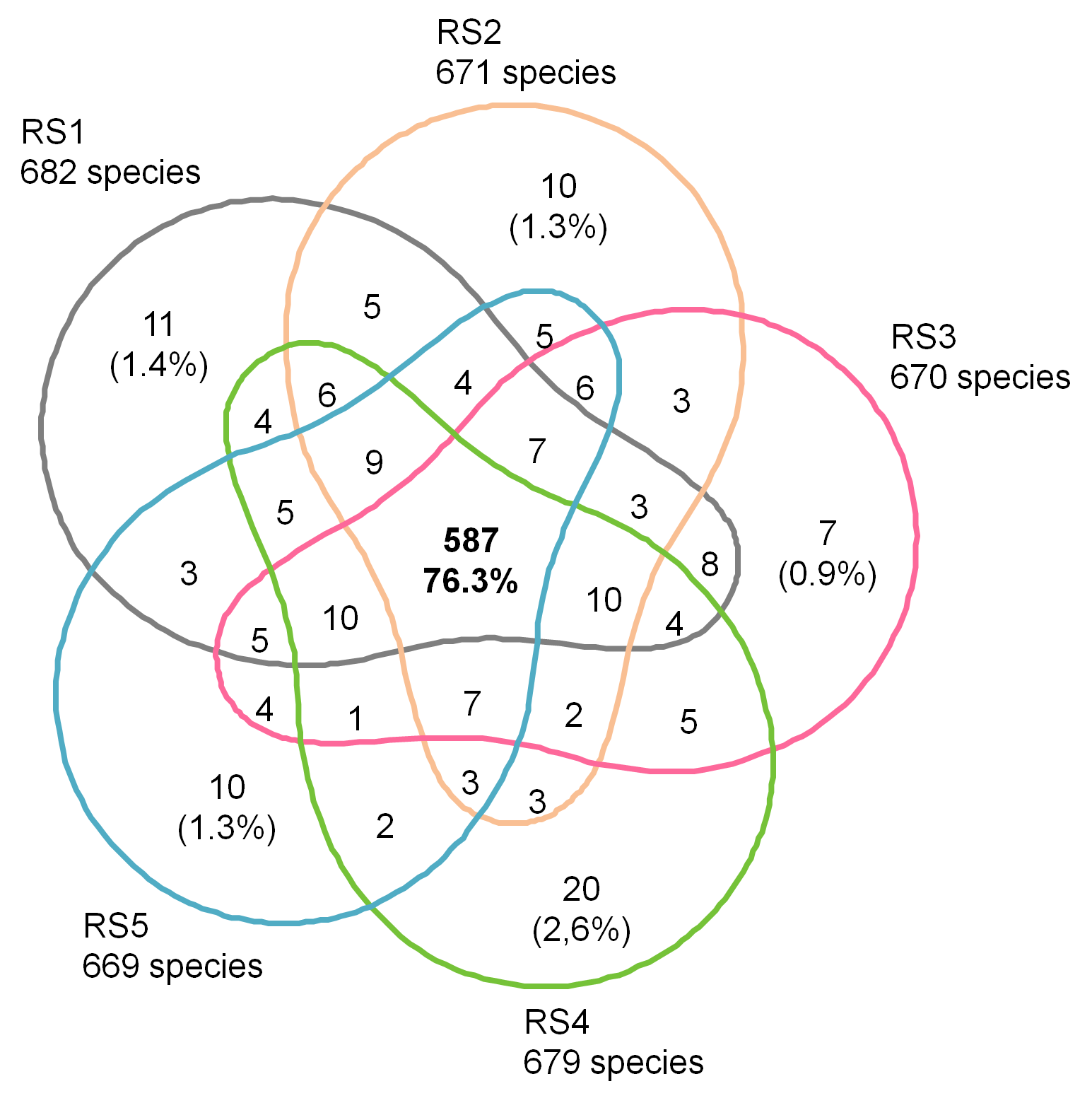


**Supplementary Fig. S4.** Five repetitive random samplings (RS) of a single random microbiomics dataset; 1.4 million reads per dataset were randomly selected out of 7 million reads that were sequenced in total. 97-99% of the species were found to be concordant across the datasets, while 0.9-2.6% were specific for a dataset. The species counts per dataset ranged from 669 to 682.

A


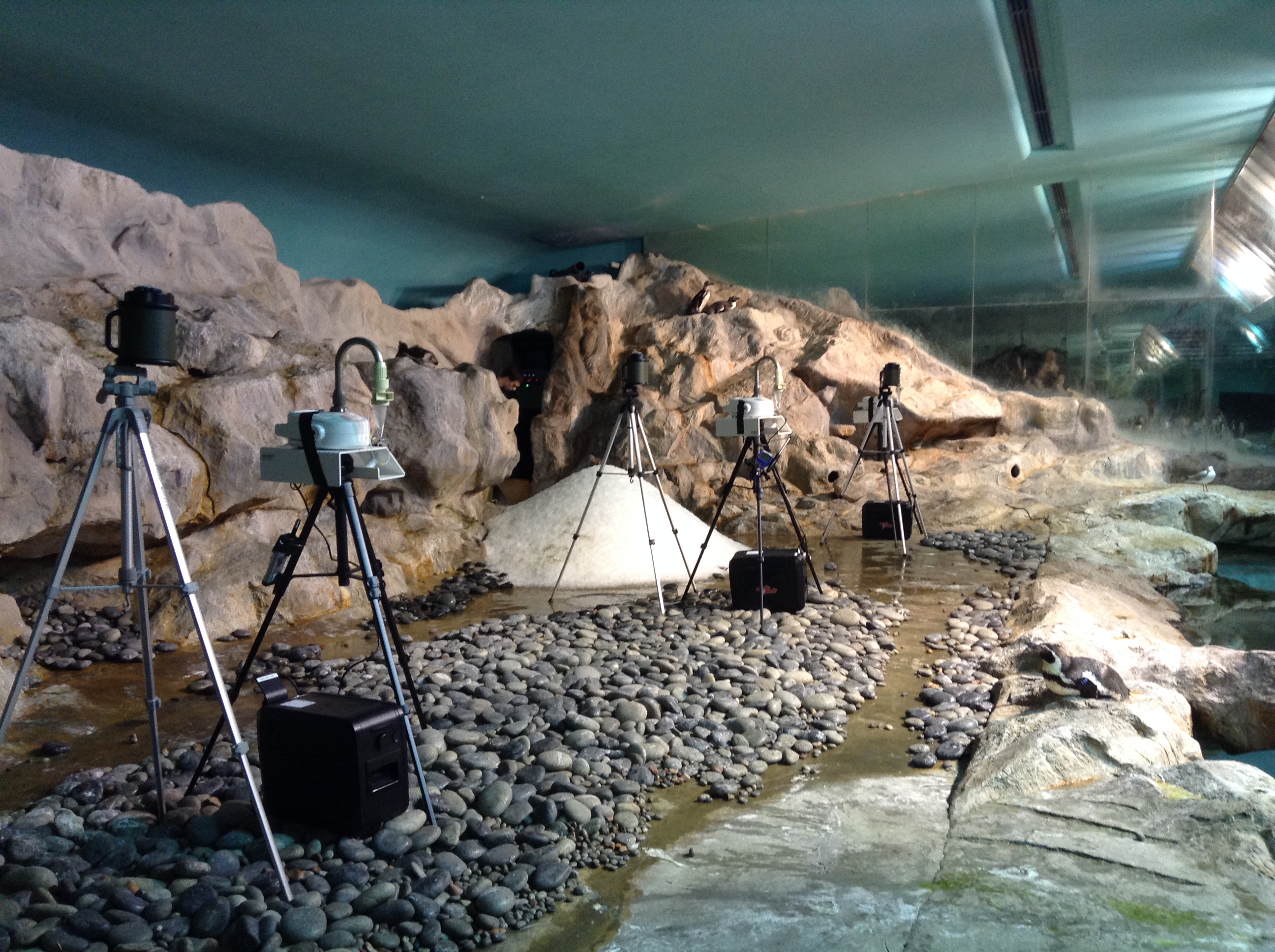


B


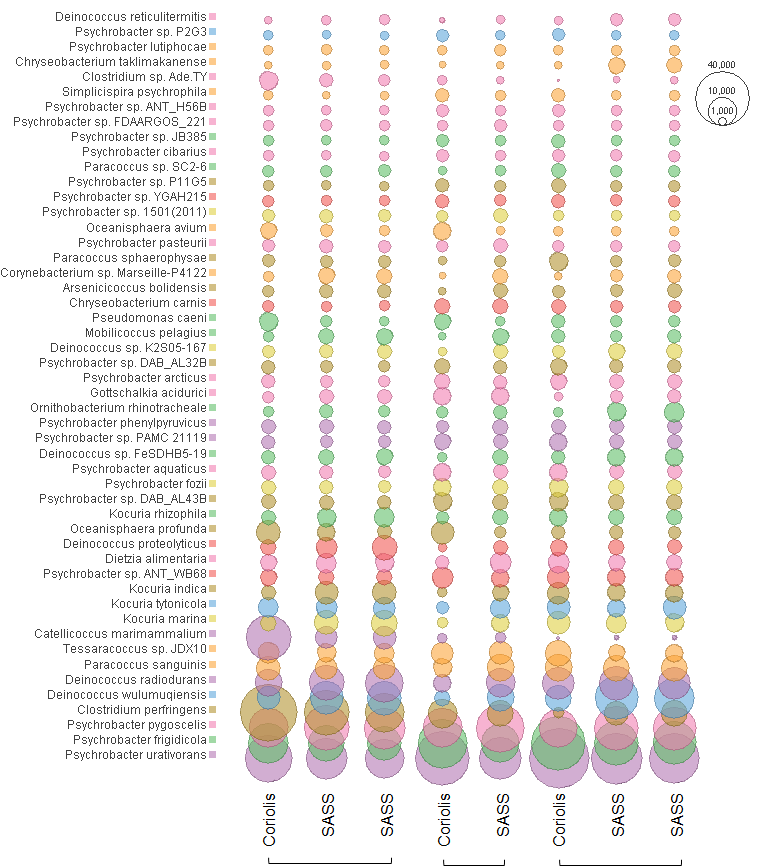


**Supplementary Fig. S5.** **A.** Sampling set up at the PE location. Filter-based SASS3100 (black) and liquid-based Coriolis Micro (white) samplers are depicted. Photo taken by Nicolas E. Gaultier **B.** Concordance between the microbiome sequencing data of samples collected using Coriolis Micro and SASS3100 air samplers. Samples were collected at the Penguin Enclosure (PE) microhabitat. The comparative analysis of various air samples including SASS and Coriolis was reported by Dybwad et al., Aerosol Science and Technology, 2014.

**Supplementary Table S1.** Random forest classification analysis. Total estimated error rate is 3.33%. Mismatches are indicated in red. PE – Penguin Enclosure, ENT – Entrance, WTL – Wetlands, LL – Lory Loft, WF – Waterfall, BG – Bridge, BP – Birds of Play.

|  | PB | BG | ENT | LL | PE | WF | WTL | Classification error |
| --- | --- | --- | --- | --- | --- | --- | --- | --- |
| PB | 16 | 0 | 0 | 0 | 0 | 2 | 0 | 0.11 |
| BG | 0 | 18 | 0 | 0 | 0 | 0 | 0 | 0 |
| ENT | 1 | 0 | 17 | 0 | 0 | 0 | 0 | 0.056 |
| LL | 0 | 0 | 0 | 17 | 0 | 1 | 0 | 0.056 |
| PE | 0 | 0 | 0 | 0 | 12 | 0 | 0 | 0 |
| WF | 0 | 0 | 0 | 0 | 0 | 18 | 0 | 0 |
| WTL | 0 | 0 | 0 | 0 | 0 | 0 | 18 | 0 |

**Supplementary Table S2.** Metagenomic analysis for filter blank samples.

| **Filter Blanks** | **Absolute read counts** |
| --- | --- |
| **Filter Blank 1** |  |
| *Klebsiella pneumoniae* | 116 |
| *Klebsiella quasipneumoniae* | 749 |
| *Polyporaceae* | 38 |
| Not assigned | 584 |
| No hits | 12152 |
| **Filter Blank 2** |  |
| *Polyporaceae* | 34 |
| Not assigned | 537 |
| No hits | 16854 |
| **Filter Blank 3** |  |
| Not assigned | 190 |
| No hits | 25306 |

**Supplementary Table S3.** Source tracking analysis results.

Species relative abundances of technical replicates (triplicates) were averaged to obtain individual observations. Sink locations: PE – Penguin Enclosure, ENT – Entrance, WTL – Wetlands, LL – Lory Loft, WF – Waterfall, BG – Bridge, BP – Birds of Play. Source locations: DVE2-5 – Day Variation Experiments 2-5.

| **Independent observations** | **Sink locations** | **Source tracking assessments** | | | | | **Standard deviations** | | | | |
| --- | --- | --- | --- | --- | --- | --- | --- | --- | --- | --- | --- |
|  |  | **DVE2** | **DVE3** | **DVE4** | **DVE5** | **Unknown** | **DVE2** | **DVE3** | **DVE4** | **DVE5** | **Unknown** |
| 1 | PE | 0.004 | 0.005 | 0.003 | 0.002 | 0.986 | 0.002 | 0.003 | 0.002 | 0.001 | 0.003 |
| 2 | PE | 0.010 | 0.003 | 0.003 | 0.002 | 0.982 | 0.002 | 0.001 | 0.002 | 0.001 | 0.003 |
| 3 | PE | 0.011 | 0.007 | 0.006 | 0.004 | 0.972 | 0.004 | 0.004 | 0.002 | 0.002 | 0.005 |
| 4 | PE | 0.009 | 0.005 | 0.005 | 0.001 | 0.980 | 0.002 | 0.002 | 0.002 | 0.001 | 0.004 |
| 5 | PE | 0.009 | 0.006 | 0.005 | 0.003 | 0.977 | 0.001 | 0.003 | 0.003 | 0.002 | 0.001 |
| 6 | PE | 0.024 | 0.037 | 0.024 | 0.014 | 0.902 | 0.006 | 0.008 | 0.005 | 0.004 | 0.010 |
| 7 | PE | 0.008 | 0.003 | 0.004 | 0.004 | 0.981 | 0.003 | 0.002 | 0.002 | 0.002 | 0.004 |
| 8 | PE | 0.005 | 0.004 | 0.003 | 0.001 | 0.988 | 0.002 | 0.002 | 0.001 | 0.001 | 0.004 |
| 9 | PE | 0.005 | 0.003 | 0.003 | 0.001 | 0.988 | 0.002 | 0.002 | 0.001 | 0.001 | 0.004 |
| 10 | PE | 0.006 | 0.005 | 0.004 | 0.003 | 0.983 | 0.002 | 0.002 | 0.002 | 0.001 | 0.003 |
| 1 | ENT | 0.062 | 0.188 | 0.442 | 0.267 | 0.040 | 0.011 | 0.019 | 0.042 | 0.026 | 0.004 |
| 2 | ENT | 0.070 | 0.245 | 0.329 | 0.208 | 0.147 | 0.008 | 0.023 | 0.033 | 0.038 | 0.002 |
| 3 | ENT | 0.226 | 0.210 | 0.260 | 0.242 | 0.063 | 0.018 | 0.022 | 0.024 | 0.038 | 0.003 |
| 4 | ENT | 0.106 | 0.145 | 0.498 | 0.189 | 0.062 | 0.014 | 0.027 | 0.041 | 0.032 | 0.004 |
| 5 | ENT | 0.090 | 0.188 | 0.286 | 0.249 | 0.187 | 0.018 | 0.010 | 0.026 | 0.021 | 0.004 |
| 6 | ENT | 0.162 | 0.168 | 0.382 | 0.225 | 0.063 | 0.016 | 0.024 | 0.036 | 0.042 | 0.003 |
| 1 | WTL | 0.106 | 0.214 | 0.305 | 0.258 | 0.118 | 0.014 | 0.024 | 0.027 | 0.022 | 0.004 |
| 2 | WTL | 0.134 | 0.165 | 0.171 | 0.122 | 0.408 | 0.011 | 0.018 | 0.018 | 0.017 | 0.004 |
| 3 | WTL | 0.141 | 0.165 | 0.142 | 0.087 | 0.465 | 0.016 | 0.016 | 0.014 | 0.019 | 0.006 |
| 4 | WTL | 0.184 | 0.233 | 0.171 | 0.142 | 0.270 | 0.021 | 0.019 | 0.024 | 0.014 | 0.007 |
| 5 | WTL | 0.128 | 0.160 | 0.130 | 0.150 | 0.432 | 0.017 | 0.020 | 0.017 | 0.020 | 0.007 |
| 6 | WTL | 0.120 | 0.126 | 0.034 | 0.064 | 0.655 | 0.016 | 0.020 | 0.007 | 0.015 | 0.008 |
| 1 | LL | 0.133 | 0.183 | 0.265 | 0.372 | 0.047 | 0.017 | 0.026 | 0.022 | 0.018 | 0.004 |
| 2 | LL | 0.198 | 0.208 | 0.239 | 0.228 | 0.127 | 0.025 | 0.017 | 0.019 | 0.026 | 0.005 |
| 3 | LL | 0.136 | 0.267 | 0.289 | 0.244 | 0.065 | 0.017 | 0.027 | 0.036 | 0.033 | 0.004 |
| 4 | LL | 0.281 | 0.267 | 0.140 | 0.126 | 0.186 | 0.024 | 0.021 | 0.015 | 0.013 | 0.006 |
| 5 | LL | 0.168 | 0.231 | 0.166 | 0.177 | 0.258 | 0.018 | 0.021 | 0.015 | 0.014 | 0.005 |
| 6 | LL | 0.187 | 0.273 | 0.178 | 0.245 | 0.117 | 0.017 | 0.011 | 0.018 | 0.022 | 0.003 |
| 1 | WF | 0.141 | 0.342 | 0.220 | 0.228 | 0.069 | 0.020 | 0.025 | 0.024 | 0.033 | 0.002 |
| 2 | WF | 0.194 | 0.242 | 0.241 | 0.252 | 0.072 | 0.021 | 0.015 | 0.025 | 0.026 | 0.004 |
| 3 | WF | 0.115 | 0.284 | 0.225 | 0.356 | 0.021 | 0.021 | 0.042 | 0.036 | 0.035 | 0.002 |
| 4 | WF | 0.215 | 0.616 | 0.078 | 0.070 | 0.022 | 0.037 | 0.026 | 0.011 | 0.018 | 0.004 |
| 5 | WF | 0.252 | 0.491 | 0.056 | 0.145 | 0.057 | 0.049 | 0.028 | 0.012 | 0.025 | 0.004 |
| 6 | WF | 0.182 | 0.355 | 0.263 | 0.140 | 0.060 | 0.016 | 0.026 | 0.023 | 0.007 | 0.004 |
| 1 | BG | 0.148 | 0.189 | 0.268 | 0.306 | 0.090 | 0.016 | 0.018 | 0.017 | 0.021 | 0.003 |
| 2 | BG | 0.128 | 0.229 | 0.207 | 0.208 | 0.228 | 0.020 | 0.027 | 0.030 | 0.026 | 0.007 |
| 3 | BG | 0.136 | 0.212 | 0.248 | 0.146 | 0.258 | 0.015 | 0.014 | 0.016 | 0.022 | 0.006 |
| 4 | BG | 0.165 | 0.198 | 0.097 | 0.038 | 0.502 | 0.021 | 0.010 | 0.012 | 0.012 | 0.008 |
| 5 | BG | 0.116 | 0.194 | 0.223 | 0.211 | 0.255 | 0.027 | 0.023 | 0.019 | 0.019 | 0.005 |
| 6 | BG | 0.215 | 0.201 | 0.190 | 0.230 | 0.165 | 0.021 | 0.025 | 0.033 | 0.024 | 0.003 |
| 1 | BP | 0.090 | 0.286 | 0.266 | 0.289 | 0.070 | 0.011 | 0.020 | 0.023 | 0.035 | 0.003 |
| 2 | BP | 0.150 | 0.209 | 0.236 | 0.269 | 0.136 | 0.019 | 0.026 | 0.030 | 0.031 | 0.004 |
| 3 | BP | 0.137 | 0.261 | 0.238 | 0.197 | 0.167 | 0.021 | 0.028 | 0.034 | 0.025 | 0.003 |
| 4 | BP | 0.187 | 0.250 | 0.122 | 0.093 | 0.348 | 0.013 | 0.013 | 0.011 | 0.010 | 0.010 |
| 5 | BP | 0.162 | 0.243 | 0.205 | 0.218 | 0.171 | 0.017 | 0.023 | 0.014 | 0.023 | 0.005 |
| 6 | BP | 0.195 | 0.144 | 0.229 | 0.400 | 0.031 | 0.031 | 0.026 | 0.029 | 0.029 | 0.005 |
